# Late presentation of congenital diaphragmatic Hernia after a diagnostic laparoscopic surgery (a case report)

**DOI:** 10.1186/1749-8090-8-8

**Published:** 2013-01-14

**Authors:** Kok Hooi Yap, Mark Jones

**Affiliations:** 1 Cardiothoracic Surgery Unit, Wythenshawe Hospital, Southmoor Road, Manchester, Wythenshawe, M23 9LT, England

**Keywords:** Congenital diaphragmatic hernia, Delayed presentation, Diagnosis, Laparoscopic surgery, Prolene mesh, Hernia repair

## Abstract

The authors report a rare case of 17-year-old lady with late presentation of congenital diaphragmatic hernia. She presented with vague abdominal pain and was thought to have urinary tract infection, ruptured ovarian cyst, and appendicitis by different medical teams in the first few days. She eventually underwent a diagnostic laparoscopy with no significant findings. In the early postoperative recovery period, she suffered from severe cardiorespiratory distress and a large intestinal left diaphragmatic hernia was diagnosed subsequently. At further operation a strangulated loop of large bowel herniating through a left antero-lateral congenital diaphragmatic hernia was discovered, which was reduced and repaired with a prolene mesh through thoracotomy. She made an excellent recovery and was discharged a few days after the operation. The authors postulate a mechanism of positive pressure from laparoscopic surgery causing herniation of large bowel through a pre-existing diaphragmatic defect. This case highlights the diagnostic challenge of this disease due to its diverse clinical presentation, the importance of prompt diagnosis and intervention.

## Background

Congenital diaphragmatic hernia (CDH) has been described since the 17^th^ century with a high mortality rate [1]. Its estimated incidence has been reported to be 1 in 2000–5000 live births [2]. CDH usually presents with respiratory distress during the neonatal period, and is associated with pulmonary hypoplasia. The prognosis depends on the degree of pulmonary hypoplasia, pulmonary hypertension and other anomalies [2].

Late presentation of CDH is uncommon, accounting for 5-30% of all CDH cases in several studies [3]. The types of CDH with late presentation include foramen of Morgagni (retrosternal defect), foramen of Bochdalek (posterolateral defect), paraesophageal hernia and eventration [3]. A history of trauma should always be considered in the presentation of diaphragmatic hernia.

We report a case of a 17-year-old girl who presented with non-specific lower abdominal pain, underwent negative laparoscopy, and deteriorated post operatively. A diagnosis of left diaphragmatic hernia was made, confirmed at operation and successfully treated.

## Case presentation

A 17-year-old lady presented to the Accident and Emergency Department with a 5-day-history of constant, sharp, localized left sided abdominal pain, urinary frequency and vomiting. Her bowel habits were normal. There were no respiratory symptoms and cardiorespiratory examination was unremarkable. Her abdominal examination revealed mild tenderness in the supra-pubic region with normal bowel sounds. She was diagnosed with urinary tract infection and was sent home with trimethoprim.

She returned the next day with no improvement of symptoms. A urinary pregnancy test was performed and showed positive results. Her chest examination remained normal and chest-x-ray (CXR) was not performed. After consulting with an obstetrician, a diagnostic laparoscopy was carried out because of suspected ruptured ovarian cyst. At laparoscopy, normal uterus and ovaries were noted and general surgical opinion was sought. Minor bowel adhesions were noted and appendicectomy was performed.

**Figure 1:**
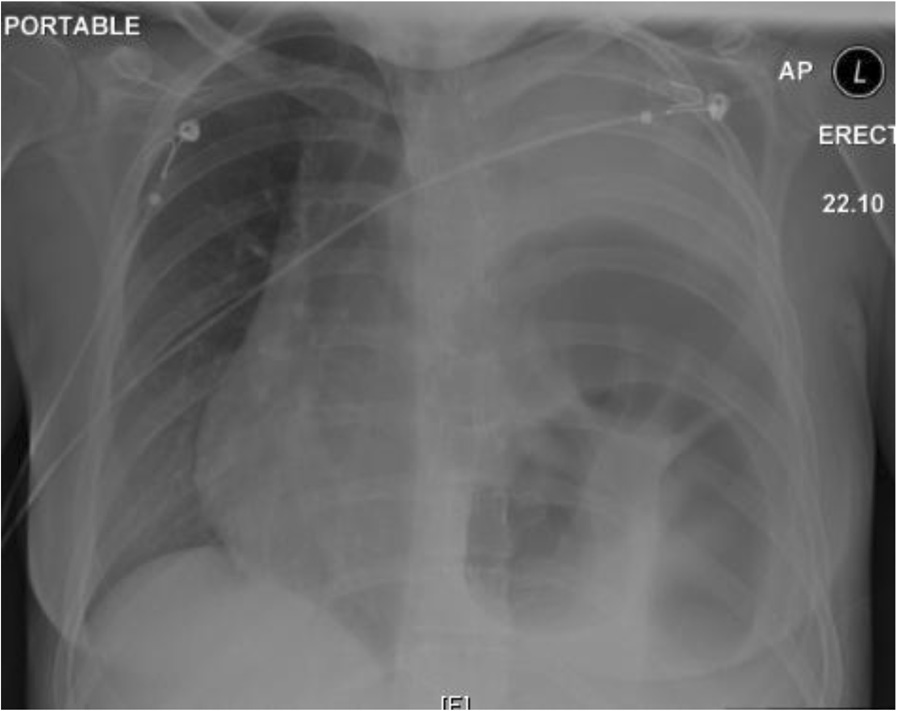
**The Chest-X-Ray performed after laparoscopic surgery.** It shows dilated bowels loops in the left hemi thorax shifting the mediastinum to the right.

**Figure 2:**
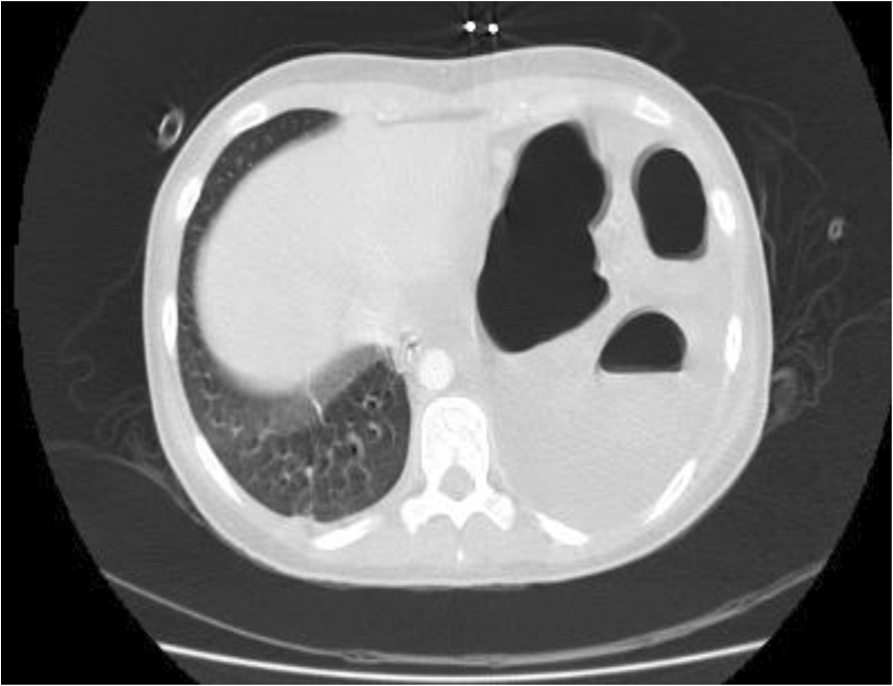
**This is the Computed Tomography of the chest and upper abdomen performed after the CXR in Figure**1**.** It shows distended bowel loops in the thorax.

Her recovery was slow postoperatively. One day after operation, she developed cardiorespiratory distress with respiratory rate of 40/minute, a documented tachycardia of 200 beats per minute and mild hypoxia. Clinical examination revealed reduced left sided air entry. A CXR was performed (Figure 1) and showed dilated large bowel loops in the left hemi thorax shifting the mediastinum to the right. A diaphragmatic hernia was diagnosed. She was managed with nasogastric tube placement, fluid resuscitation, and supportive treatment in the Intensive Care Unit. A Computed Tomography (CT) of her chest and upper abdomen was completed (Figure 2). It showed left pleural effusion, lung atelectasis and distended bowel loops with a fluid level in the thorax. This was consistent with a diaphragmatic hernia with bowel obstruction.

She was transferred to a Cardiothoracic Surgical Unit for urgent surgical intervention. A left postero-lateral thoracotomy approach was used. Copious clear serous fluid was present in the chest, and there was a strangulated loop of large intestines herniated through a small left antero-lateral congenital diaphragmatic defect approximately 3cm X 5cm. The defect was expanded through an incision on the diaphragm to facilitate the reduction of the bowel into the abdominal cavity which was then repaired with a prolene mesh. Her left lung was noticed to be normal.

**Figure 3:**
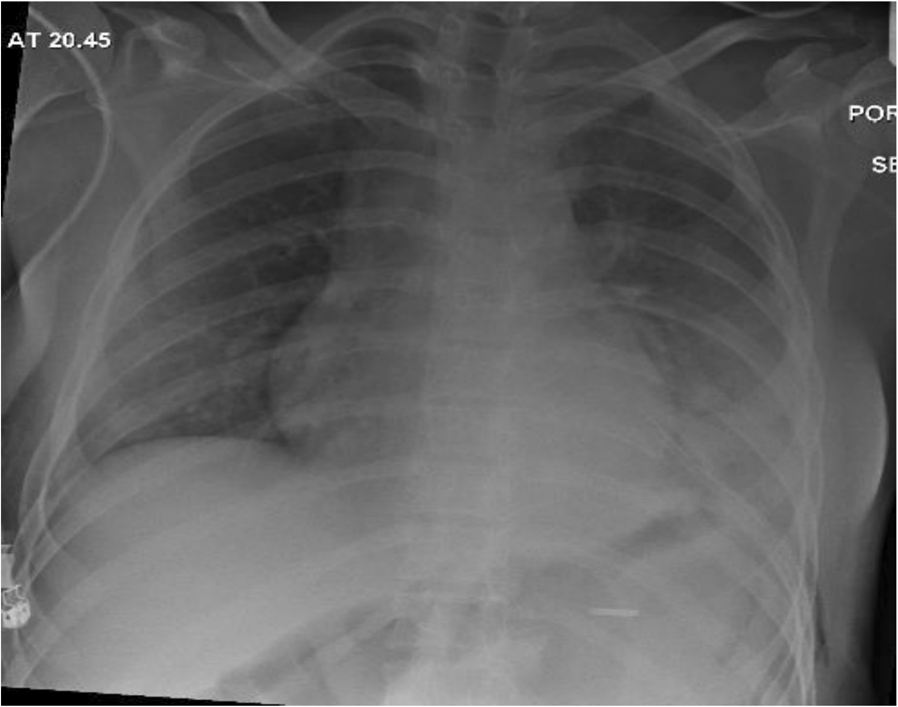
**This is the CXR after the diaphragmatic hernia repair.** It shows that the bowel contents have been successfully reduced with satisfactory left lung expansion.

She was discharged to the ward after 24-hour care in critical care unit. Figure 3 shows her post-operative CXR. A smooth recovery was achieved and she went home a few days later. She was then followed-up by the obstetric team and two months later her pregnancy remained viable.

## Discussion

Beyond infancy diaphragmatic hernia is an unusual finding. There are different causes other than CDH, for example, trauma, phrenic nerve palsy, and longstanding acquired hiatus hernia.

The Congenital Diaphragmatic Hernia Study Group conducted a 10-year study across 30 centers to review the demographics, clinical manifestation and outcomes of late onset CDH [4]. Of 3098 cases of CDH, only 79 patients presented with late-onset (2.6%). The male to female ratio was 1.8 with mean age at diagnosis of 372 days. This emphasizes the rarity of the case as our patient was female with age of 17 at presentation [4].

Presenting symptoms of late onset CDH could be classified as respiratory (upper respiratory tract infection, pneumonia, respiratory distress, cough, wheezing, etc.), gastrointestinal (vomiting, abdominal pain, failure to thrive, constipation, etc.), both or asymptomatic [4]. Gastrointestinal symptoms predominate in left sided hernias whereas respiratory symptoms are more common in right-sided lesions [4].

The group speculates that partial liver herniation in right CDH impedes further herniation of hollow organs into the chest, thereby obviating gastrointestinal symptoms [1,4]. Interestingly, the patient in this report was found to have a mild enlarged spleen. This could have contributed to the late presentation of her CDH.

The CDH Study Group also concluded that the prognosis of late-onset CDH is excellent once the correct diagnosis is made. However, making the correct diagnosis is challenging because of its diverse clinical presentations. Clinical suspicion and CXR remain key. Nevertheless, the variety of organs and the size of herniation may make CXR interpretation difficult and late presenting CDH may mimic pneumonia, tumor and diaphragmatic eventration. The hernia may be intermittent and the presence of a normal CXR does not exclude the diagnosis [5]. Further imaging evaluation is warranted if clinical suspicion is high e.g. contrast series or CT.

It was difficult to make the correct diagnosis at the initial stage of this patient because of the paucity of respiratory symptoms and absent chest signs. Imaging was complicated by the early pregnancy. The cause of her initial vague lower abdominal pain was difficult to ascertain leading to laparoscopy. It is possible that the initial diagnostic laparoscopy missed either the early stage or an established herniation of large bowel through the defect. We believe, however, that the positive pressure of the diagnostic laparoscopy might have pushed the hollow organs through the diaphragmatic defect hence allowing it to present itself. This has only once been reported in the past [6].

Following diagnosis the management of acute presentation of CDH aims to achieve urgent bowel decompression. This is critical to prevent complications such as gastric volvulus, bowel strangulation or respiratory complications. In this case the herniated large bowel was strangulated with profound venous engorgement, which could have led to infarction.

The timing of surgery, different types of surgical approach and repair techniques have been greatly studied but this will not be discussed any further in this report.

## Conclusion

This case highlights the diagnostic difficulties that clinicians face when dealing with late-onset CDH due to its rarity and diverse presentations. It emphasizes the importance of the sound clinical evaluation, correct interpretation of imaging, and consideration of CDH in all age groups when clinical suspicion is raised. Extensive search of the literature demonstrates that this as the first reported case of late presenting strangulated CDH, due to positive pressure from laparoscopic surgery.

## Consent

Written informed consent was obtained from the patient for publication of this case report and accompanying images. A copy of the written consent is available for review by the Editor-in-Chief of this journal.

## Abbreviations

CDH: Congenital Diaphragmatic Hernia; CT: Computerized Tomography; CXR: Chest-X-Ray.

## Competing interests

The authors declare that they have no competing interests.

## Authors’ contributions

KHY contributed in pre-operative, intraoperative and post-operative care. He also collected data and prepared the first draft. MTJ contributed in pre-operative, intraoperative and post-operative care. He also revised the article thoroughly and critically. Both authors approved the final version to be published.

## References

[B1] Chao PH Chuang JH Lee SY Huang HC Late-presenting congenital diaphragmatic hernia in childhood Acta Paediatr 2011 100 3 425 428 10.1111/j.1651-2227.2010.02025.x 20874742

[B2] Kesieme EB Kesieme CN Congenital Diaphragmatic Hernia: review of current concept in surgical management ISRN Surg 2011 2011 974041 22229104 10.5402/2011/974041 PMC3251163

[B3] Singh S Bhende MS Kinnane JM Delayed presentations of congenital diaphragmatic hernia Pediatr Emerg Care 2001 17 4 269 271 10.1097/00006565-200108000-00013 11493830

[B4] Kitano Y Lally KP Lally PA Congenital Diaphragmatic Hernia study group: Late-presenting congenital diaphragmatic hernia J Pediatr Surg 2005 40 12 1839 1843 16338301 10.1016/j.jpedsurg.2005.08.023

[B5] Lee HM Addavide KE Prince NJ Late presentation of a diaphragmatic hernia Arch Dis Child 2011 96 9 837 10.1136/archdischild-2011-300287 21693506

[B6] Jai SR Boufettal R Cherkab R Chehab F Khaiz D Barrou H Bouzidi A Diaphragmatic hernia as an unusual cause of respiratory distress following laparoscopic surgery J Chir (Paris) 2009 146 3 325 327 10.1016/j.jchir.2009.06.006 19640534

